# Alcohol Consumption and Risk of Chronic Kidney Disease: A Nationwide Observational Cohort Study

**DOI:** 10.3390/nu11092121

**Published:** 2019-09-06

**Authors:** Yun-Ju Lai, Yu-Yen Chen, Yu-Kai Lin, Chu-Chieh Chen, Yung-Feng Yen, Chung-Yeh Deng

**Affiliations:** 1 Division of Endocrinology and Metabolism, Department of Internal Medicine, Puli Branch of Taichung Veterans General Hospital, Nantou 54552, Taiwan; 2 School of Medicine, National Yang-Ming University, Taipei 11221, Taiwan; 3 Department of Exercise Health Science, National Taiwan University of Sport, Taichung 40404, Taiwan; 4 Department of Ophthalmology, Taichung Veterans General Hospital, Taichung 40705, Taiwan; 5 Department of Health and Welfare, College of City Management, University of Taipei, Taipei 11153, Taiwan; 6 Department of Health Care Management, National Taipei University of Nursing and Health Sciences, Taipei 11219, Taiwan; 7 Section of Infectious Diseases, Taipei City Hospital, Taipei City Government, Taipei City Government, No.145, Zhengzhou Road, Datong District, Taipei City 10341, Taiwan; 8 Institute of Hospital and Health Care Administration, National Yang-Ming University, 155, Section 2, Ni-Long Street, Taipei 11221, Taiwan

**Keywords:** alcohol, cohort study, chronic kidney disease

## Abstract

Alcohol consumption is a significant public health issue worldwide. The rat model and epidemiological studies have both reported conflicting results about the effects of alcohol on the kidneys. We aimed to explore the relationships between alcohol consumption and chronic kidney disease. Data from the National Health Interview Survey, the National Health Insurance research database, and the National Deaths Dataset were used. Standardized in-person interviews were executed in 2001, 2005, and 2009 to obtain the demographic characteristics of study population. The participants were followed up until 2013. The primary outcome was new-onset chronic kidney disease. We analyzed 45,200 adults older than 18 years (50.8% men and 49.2% women), and the overall mean (SD) age was 42.73 (16.64) years. During the 8.5 (3.5) years of follow-up, new-onset chronic kidney disease was recognized in 1535 (5.5%), 292 (2.7%), and 317 (4.9%) non-drinking, social-drinking, and regular-drinking participants, respectively. The participants who were social and regular drinkers had a significantly decreased risk of chronic kidney disease incidence (social drinking: adjusted hazard ratio (HR), 0.85; 95% confidence interval (CI), 0.74–0.97; *p* = 0.018; regular-drinking: AHR, 0.85; 95% CI, 0.74–0.98; *p* = 0.024), with baseline demographics and comorbidities adjusted. In conclusion, social and regular drinkers had decreased risk of chronic kidney disease when compared with non-drinkers.

## 1. Introduction

Alcohol consumption is a significant global public health issue. Moderate alcohol consumption is associated with a lower risk of cardiovascular complications [1], whereas abundant alcohol consumption is a well-confirmed risk factor for hepatitis, pancreatitis, cardiovascular events, and cancer [2,3]. However, the influence of alcohol on kidney morphology and performance remains poorly understood.

Animal studies on this topic had conflicting results. Ethanol administration in rats showed particular alterations in the renal antioxidant system and glutathione status [4,5]. Polyphenols, which are found in beverages, such as red wine, also have antioxidant effects [6,7]. However, another rat model showed that ethanol may increase blood pressure and angiotensin II type 1 receptor expression, causing glomerular morphology changes. This may lead to renal corpuscle and glomeruli atrophy and reduced glomeruli volume [8].

In epidemiological research, the link between alcohol consumption and kidney function remains inconclusive. Several cross-sectional research has shown negative correlations between drinking and the prevalence of chronic kidney disease (CKD) [9,10,11]. Studies from Taiwan, Japan, and Korea reported that alcohol consumption was reversely associated with the existence of CKD [9,10,11]. A cross-sectional study in Taiwan analyzed 15,353 women and 11,900 men in middle-age. The result showed that alcohol consumption was negatively associated with the presence of stage 3 CKD only in men [9]. Another cross-sectional study in Korean with 5251 participants revealed that alcohol consumption was inversely associated with a reduction in the estimated glomerular filtration rate in men [10]. In Japan, a cross-sectional study analyzed 292,013 individuals that were aged more than 40 years and reported that mild to moderate alcohol drinking was associated with a lower prevalence of proteinuria [11]. However, causality is poorly demonstrated in cross-sectional studies. Several cohort studies have also reported diverse results. A population-representative cohort study in Australia indicated that daily alcohol consumption was correlated to a higher risk of albuminuria, but a lower risk of estimated glomerular filtration rate eGFR <60 mL/min/1.73 m^2^ [12]. A community-based cohort study in the USA investigated the effect of alcohol on kidneys in an elderly population (>65 years old) and found no significant association between the quantity of weekly ethanol consumption and kidney function deterioration [13]. The quantity of alcohol that was consumed weekly was related to a lower risk of developing CKD in a population-based cohort study in the Netherlands [14]. Based on these conflicting results, the impact of alcohol on the kidneys remains inconclusive. Thus, we used data that were representative of the Taiwan population to explore the relationships between alcohol intake and development of CKD.

## 2. Materials and Methods

### 2.1. Data Collection

We used data from the National Health Interview Survey (NHIS) in 2001, 2005, and 2009; the National Health Insurance research database; and, the National Deaths Dataset. All data were composed, organized, and explored in the Health and Welfare Data Science Center of Ministry of Health and Welfare in Taiwan. The National Health Interview Survey selected participants while using a multistage stratified systematic sampling design. Participant information, including education, income, marriage status, and lifestyle behaviors, were obtained during in-person interviews. The National Health Insurance research database comprises medical information of nearly 99% of Taiwanese people, including ambulatory and inpatient care.

### 2.2. Ethical Statement

The Taipei City Hospital Institutional Review Board permitted this research (TCHIRB-10709107-W). Informed consent was obtained from all patients.

### 2.3. Alcohol Consumption

Information of alcohol consumption was obtained by asking three questions: “Do you currently drink alcohol?”, “How often do you drink?”, and “Do you usually get drunk?” The main exposure variable, alcohol consumption, was then categorized into three categories: non-drinker (currently no drinking), social drinker (less than once a week), and regular drinker (more than once a week, including both inebriated or not) [15,16].

### 2.4. Determination of CKD

Comorbidities were recognized from the health care insurance information. Disease diagnosis in the National Health Insurance research database was coded with the International Classification of Disease, 9th Revision Clinical Modification (ICD-9-CM). The participants were considered to have a comorbidity if the diagnosis code presented in three or more outpatient claims or one hospitalization claim. The dependent variable of this research was CKD (ICD-9-CM codes 580-589) [17]. 

### 2.5. Potential Confounders

Participant information, including socioeconomic status, marriage status, education level, and health behaviors, was collected by well experienced interviewers. Information regarding fruit and vegetable consumption was collected by the questions “How many days do you eat fruit in a week?” and “How many days do you eat vegetables in a week?” The answers were “never, <1 time, 1–2 times, 3–5 times, and daily”. Obesity was determined as body mass index that was greater than 27 kg/m^2^ [18], as suggested by the Health Promotion Administration of Ministry of Health and Welfare. Information regarding physical activity during leisure time was estimated while using the calculation: activity intensity code (kcal/min) × frequency per week (times) × duration for each time (minutes) [19]. Comorbidities were documented from the diagnosis codes of the National Health Insurance research database, including diabetes mellitus (ICD-9-CM code 250), hypertension (ICD-9-CM codes 401-405), urolithiasis (ICD-9-CM codes 592.0, 592.1, 594.0, 594.1), and gouty arthritis (ICD-9-CM code 274).

### 2.6. Study Design

This was a nationwide prospective cohort study. Subjects that were aged more than 18 years old were selected from the 2001, 2005, and 2009 NHIS. Those with a diagnosis of CKD in the medical insurance record before the interview date were excluded. The follow-up duration began since the interview date and censored on the date of incident CKD, death, or Dec 31, 2013, which ever come first.

### 2.7. Statistical Analysis

CKD survival curves were drawn by means of the Kaplan–Meier method with differences across the three categories of alcohol consumption being assessed by the log-rank test. We used non-drinkers as the reference group to calculate the hazard ratios (HRs) and 95% confidence intervals (CIs) for CKD by univariable and multivariable Cox proportional-hazards regression models. The following variables were adjusted in the multivariable Cox models: age, gender, education, weekly intake of vegetables and fruit, physical activity during leisure time, and comorbidities, including diabetes mellitus, hypertension, urolithiasis, and gouty arthritis. Data analyses were generated in SAS 9.4 (SAS Institute, Cary, North Carolina, NC, USA).

## 3. Results

**Figure 1:**
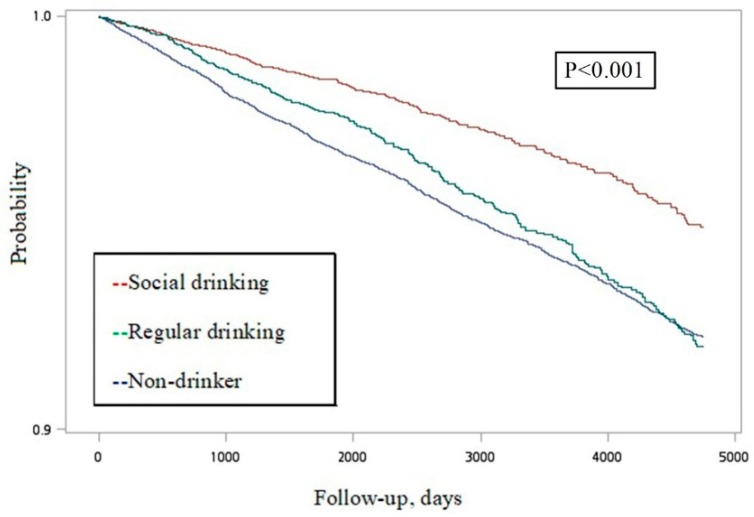
Kaplan–Meier survival curve estimates for incident chronic kidney disease in a random community sample from Taiwan.

There were 48,604 adults older than 18 years that participated in the three rounds of the NHIS in 2001, 2005, and 2009. After excluding those already diagnosed with CKD (*n* = 1365), unidentified sex (*n* = 1), and unavailable information on alcohol consumption (*n* = 1918), there were 45,200 participants that were enrolled in the investigation. Among them, 22,971 were male (50.8%) and the mean (SD) age was 42.73 (16.64) years old. The follow-up period was 8.5 (3.5) years. Figure 1 presents the Kaplan–Meier curves of the three categories of alcohol consumption, which indicated a significant difference (*p* < 0.001).

Table 1 shows the result of the univariable Cox regression model to examine the baseline characteristics and comorbidities of study participants. Regarding the alcohol consumption categories, 27,781 (61.5%), 10,997 (24.3%), and 6422 (14.2%) of participants were categorized as non-drinkers, social drinkers, and regular drinkers, respectively. During 384,502 person-years of follow-up, new-onset CKD was recognized in 1535 (5.5%), 29 (2.7%), and 317 (4.9%) participants who were non-drinkers, social-drinkers, and regular-drinkers, respectively. When compared with non-drinkers, participants who were social and regular drinkers had decreased risks of CKD (social drinking: hazard ratio (HR), 0.57; 95% confidence interval (CI), 0.50–0.65; *p* < 0.001; regular-drinking: HR, 0.93; 95% CI, 0.82–1.05; *p* = 0.24). Older age, male gender, widowed/divorced/separated, obesity, and former smokers were correlated with a higher risk of incident CKD. The univariable Cox model revealed that subjects with diabetes mellitus, hypertension, hyperlipidemia, urolithiasis, and gouty arthritis had increased risk of incident CKD (*p* < 0.001).

Table 2 shows the result of the multivariable Cox regression model to recognize independent risk factors for the occurrence of CKD. The main exploratory variable is alcohol consumption, and the controlled variables included age, gender, education, vegetable, fruit, physical activity, obesity, smoking status, diabetes, hypertension, urolithiasis, and gouty arthritis. After controlling for potential confounders, those with social and regular drinking habits had significantly decreased risks of incident CKD (social drinking: (AHR), 0.85; 95% CI, 0.74–0.97; *p* = 0.018; regular-drinking: AHR, 0.85; 95% CI, 0.74–0.98; *p* = 0.024). In addition, a significant dose-response association between alcohol drinking and a decreased risk of CKD was found while using the trend test (*p* for trend = 0.009). Other variables that increased the risk of incident CKD included age, male gender, obesity, former smoking, diabetes mellitus, hypertension, urolithiasis, and gouty arthritis. Factors that were associated with decreased risk of incident CKD were high education level and vegetable intake more than five days a week.

## 4. Discussion

This 13-year cohort study revealed that participants with social or regular drinking habits had significantly reduced risk of the development of CKD when compared with non-drinkers.

The impact of alcohol on kidney function has not been well investigated. There are several possible protective mechanisms of alcohol on kidney function. Ethanol and polyphenol both have anti-oxidative effects and ethanol improves polyphenol absorption, thereby contributing to bioavailability [4,5,6]. Furthermore, alcohol has an anti-inflammatory effect, with increased serum interleukin-10 levels and decreased serum interleukin-16 levels [20]. Alcohol consumption can raise high-density lipoprotein cholesterol concentration [21,22], improve insulin sensitivity [23], and reduce platelet aggregation rate and fibrinolysis [21,22]. Alcohol consumption, including vodka and red wine, also reduced serum insulin concentrations and enhanced the insulin sensitivity index [24,25]. A moderate amount of alcohol drinking decreases the risk of developing diabetes, showing a U-shaped association [26]. 

Prior epidemiological research had shown a roughly negative relationship between alcohol consumption and the risk of CKD. Some cross-sectional studies have reported that alcohol drinking was inversely associated with prevalence of CKD. 

Matsumoto et al. analyzed data from 292,013 subjects aged more than 40 years who joined in a health checkup between 2008 and 2009 in Japan. The study indicated that alcohol consumption was inversely related with the prevalence of CKD, defined as eGFR < 60 mL/min/1.73 m^2^ [11]. However, nearly 60% of participants were omitted from the statistical analysis owing to missing data, which may lead to selection bias. Hsu et al. analyzed information from a health checkup database in Taiwan during 2003 and 2009. The cross-sectional study disclosed that alcohol consumption was inversely related with existence of stage 3 CKD in Taiwanese males [9]. Nevertheless, the frequency of alcohol consumption was obtained while using a self-reported questionnaire and the amount consumed was not available. Kim et al. analyzed data from a cross-sectional health and nutrition survey in Korea in 2011 and reported that mean daily alcohol intake was reversely associated with a reduction in eGFR in Korean males [10]. However, albuminuria might be the initial indicator of renal dysfunction, not eGFR. 

Prior cohort studies have revealed diverse results regarding the association between alcohol consumption and development of CKD. A five-year population-representative cohort study in Australia enrolled 6259 adults aged more than 25 years from 1999 until 2005. The results showed that daily alcohol consumption was associated with an increased risk albuminuria development, but a reduced risk of eGFR < 60 mL/min/1.73 m^2^ [12]. The frequency and quantity of alcohol intake was composed while using a self-administered validated food frequency questionnaire. Heavy drinkers may have selective reporting and under-reporting, which could lead to bias. Another prospective community-based cohort study in the USA enrolled 4343 subjects aged ≥65 years. The results showed no significant association between weekly alcohol intake and kidney function deterioration, which was defined as eGFR reduction more than 3 mL/min/1.73 m^2^ per year [13]. The generality of the study is limited, because only elderly individuals were investigated. Koning et al. performed a prospective nationwide cohort study in Netherlands, including 5476 subjects aged 28–75 years enrolled in 1997 and followed until 2012. The results revealed that the quantity of alcohol consumed per week was inversely associated with a decreased risk of incident CKD, which was defined as eGFR less than 60 mL/min/1.73 m^2^ or microalbuminuria [14]. The alcohol classification did not distinguish non-drinkers and former drinkers and the beverage type was not available. 

There were several limitations that should be mentioned. The data set did not contain laboratory data and the CKD diagnosis was dependent on the ICD-9-CM code. However, the possibility of misclassification was low, because the National Health Insurance overlays more than 97% of the Taiwan population and all of the medical records must be uploaded and then reviewed by the National Health Insurance Administration with high accuracy. Participants’ baseline characteristics, including weight, height, education, marriage status, household income, smoking, drinking, diet, and exercise habits, were self-reported, and recall bias should be concerned. The survey questions did not distinguish non-drinkers and former drinkers, and former drinkers were categorized as non-drinkers. Former drinkers are mostly remarkable, as their health status may be worse, and morbidity and mortality are higher than never drinkers [27]. In addition, the beverage type and exact amount of alcohol consumed were not available in the dataset. However, previous studies have not revealed beverage-specific associations [28]. Figure 1 showed the crude follow up condition of the three drinking groups. The detailed differences among the three drinking groups are analyzed by the univariable and multivariable Cox model. In the univariable Cox model, it may not meet the proportional hazard assumption. Not only the univariable analysis, but also the non-proportionality, may affect the multivariable model.

The current study enrolled 45,200 subjects, which was a representative cohort for the Taiwan population. The follow-up period was 13 years, which was long enough to observe the development of CKD; thus, the research had appropriate power. Furthermore, we adjusted for nearly all potential confounding variables, including age, sex, body mass index, socioeconomic status, vegetables, fruit, smoking, and exercise habits, and comorbidities.

## 5. Conclusions

Our study revealed that alcohol consumption was associated with a decreased risk of incident CKD. The mechanism by which alcohol influences the progress of CKD remains uncertain. Further study is warranted to investigate the influence of alcohol on the pathogenesis of CKD. However, drinking in excess of the recommended limits is not suggested for CKD prevention due to harmful health effects.

## Figures and Tables

**Table 1:** Characteristics and results of the univariable Cox regression analysis of a random community sample in Taiwan (*n* = 45,200; 2144 chronic kidney disease (CKD) cases).

Demographics	Mean ± SD/Numbers (% In Column)	Number of CKD Cases (% In Row)	Hazard Ratio	(95% CI)
Alcohol				
Non-drinker	27,781 (61.5)	1535 (5.5)	Ref	
Social	10,997 (24.3)	292 (2.7)	0.57	(0.50–0.65)
Regular	6422 (14.2)	317 (4.9)	0.93	(0.82–1.05)
Age in years, mean (SD)	42.73 (16.64)	50.08 (15.78)	1.07	(1.06–1.07)
Gender				
Female	22,971 (50.8)	960 (4.2)	Ref	
Male	22,229 (49.2)	1184 (5.3)	1.27	(1.16–1.38)
Marriage status				
Married/cohabiting	27,295 (60.4)	1523 (5.6)	Ref	
Never married	13,152 (29.1)	177 (1.4)	0.24	(0.20–0.28)
Widowed/divorced/separated	4743 (10.5)	444 (9.4)	1.96	(1.77–2.18)
Education				
Low (elementary or below)	11,108 (24.6)	1244 (11.2)	Ref	
Moderate (junior/senior high)	20,009 (44.3)	638 (3.2)	0.26	(0.24–0.29)
High (college or above)	14,046 (31.1)	256 (1.8)	0.16	(0.14–0.18)
Household income				
<US $952/month	9796 (23.2)	763 (7.8)	Ref	
US $952–2222/month	18,353 (43.4)	774 (4.2)	0.49	(0.44–0.54)
>US$ 2222/month	14,118 (33.4)	480 (3.4)	0.39	(0.34–0.43)
Obesity				
No	36,270 (84.3)	1367 (3.8)	Ref	
Yes	6771 (15.7)	474 (7.0)	1.97	(1.78–2.19)
Smoking status				
Never	31,082 (68.8)	1393 (4.5)	Ref	
Current	11,456 (25.4)	544 (4.8)	1.05	(0.95–1.16)
Former	2639 (5.8)	207 (7.8)	2.23	(1.92–2.58)
Vegetables				
<5 days/week	6358 (14.1)	330 (5.2)	Ref	
5–7 days/week	38,790 (85.9)	1808 (4.7)	0.97	(0.86–1.09)
Fruit				
<5 days/week	16,782 (37.2)	876 (5.2)	Ref	
5–7 days/week	28,365 (62.8)	1263 (4.5)	0.94	(0.86–1.02)
Physical activity				
0 kcal/week	20,963 (47.5)	976 (4.7)	Ref	
0–800 kcal/week	12,253 (27.8)	465 (3.8)	0.84	(0.75–0.94)
>800 kcal/week	10,931 (24.8)	606 (5.5)	1.24	(1.12–1.37)
Diabetes				
No	38,864 (86.0)	1292 (3.3)	Ref	
Yes	6336 (14.0)	852 (13.5)	4.15	(3.80–4.52)
Hypertension				
No	32,260 (71.4)	671 (2.1)	Ref	
Yes	12,940 (28.6)	1473 (11.4)	5.53	(5.06–6.06)
Hyperlipidemia				
No	35,395 (78.3)	1222 (3.5)	Ref	
Yes	9805 (21.7)	922 (9.4)	2.64	(2.43–2.88)
Urolithiasis				
No	42,440 (93.9)	1917 (4.5)	Ref	
Yes	2760 (6.1)	227 (8.2)	1.79	(1.56–2.05)
Gouty arthritis				
No	40,402 (89.4)	1504 (3.7)	Ref	
Yes	4798 (10.6)	640 (13.3)	3.55	(3.23–3.89)

Abbreviations: SD, standard deviation; CI, confidence interval.

**Table 2:** Results of the multivariable Cox proportional hazards analysis of the incidence of chronic kidney disease.

Demographics	Adjusted Hazard Ratio	(95% CI)	*p*-Value
Alcohol *			
Non-drinker	Ref		
Social	0.85	(0.74–0.97)	0.018
Regular	0.85	(0.74–0.98)	0.024
Age in years	1.05	(1.05–1.05)	<0.001 *
Gender			
Female	Ref		
Male	1.28	(1.14–1.44)	<0.001
Education			
Low (elementary or below)	Ref		
Moderate (junior/senior high)	0.86	(0.76–0.97)	0.013
High (college or above)	0.72	(0.61–0.84)	<0.001
Vegetable			
<5 days/week	Ref		
5–7 days/week	0.82	(0.71–0.95)	0.007
Fruit			
<5 days/week	Ref		
5–7 days/week	0.98	(0.88–1.09)	0.73
Physical activity			
0 kcal/week	Ref		
0–800 kcal/week	0.89	(0.79–1.01)	0.07
>800 kcal/week	0.92	(0.82–1.03)	0.16
Obesity (BMI ≥ 27 kg/m^2^)			
No	Ref		
Yes	1.31	(1.17–1.47)	<0.001
Smoking status			
Never	Ref		
Current	1.11	(0.97–1.26)	0.13
Former	1.3	(1.09–1.54)	0.005
Diabetes			
No	Ref		
Yes	1.65	(1.48–1.83)	<0.001
Hypertension			
No	Ref		
Yes	1.54	(1.36–1.74)	<0.001
Urolithiasis			
No	Ref		
Yes	1.19	(1.02–1.39)	0.027
Gouty arthritis			
No	Ref		
Yes	1.76	(1.58–1.96)	<0.001

The dose-response relationship between alcohol consumption and incident chronic kidney disease was evaluated using the trend test (*p* for trend = 0.009). Abbreviations: CI, confidence interval. * Adjusted Hazard Ratio = 1.049; 95% CI, 1.045–1.053; *p* < 0.001.

## References

[1] Ronksley P.E., Brien S.E., Turner B.J., Mukamal K.J., Ghali W.A. (2011). Association of alcohol consumption with selected cardiovascular disease outcomes: A systematic review and meta-analysis. BMJ (Clin. Res. Ed.).

[2] Kristiansen L., Gronbaek M., Becker U., Tolstrup J.S. (2008). Risk of pancreatitis according to alcohol drinking habits: A population-based cohort study. Am. J. Epidemiol..

[3] Varela-Rey M., Woodhoo A., Martinez-Chantar M.L., Mato J.M., Lu S.C. (2013). Alcohol, DNA methylation, and cancer. Alcohol Res. Curr. Rev..

[4] Dinu D., Nechifor M.T., Movileanu L. (2005). Ethanol-induced alterations of the antioxidant defense system in rat kidney. J. Biochem. Mol. Toxicol..

[5] Rodrigo R., Miranda A., Vergara L. (2011). Modulation of endogenous antioxidant system by wine polyphenols in human disease. Clin. Chim. Acta.

[6] Rodrigo R., Rivera G., Orellana M., Araya J., Bosco C. (2002). Rat kidney antioxidant response to long-term exposure to flavonol rich red wine. Life Sci..

[7] Rodrigo R., Castillo R., Carrasco R., Huerta P., Moreno M. (2005). Diminution of tissue lipid peroxidation in rats is related to the in vitro antioxidant capacity of wine. Life Sci..

[8] Leal S., Ricardo Jorge D.O., Joana B., Maria S.S., Isabel S.S. (2017). Heavy Alcohol Consumption Effects on Blood Pressure and on Kidney Structure Persist After Long-Term Withdrawal. Kidney Blood Press. Res..

[9] Hsu Y.H., Pai H.C., Chang Y.M., Liu W.H., Hsu C.C. (2013). Alcohol consumption is inversely associated with stage 3 chronic kidney disease in middle-aged Taiwanese men. BMC Nephrol..

[10] Kim H.N., Kim S.H., Song S.W. (2014). Is alcohol drinking associated with renal impairment in the general population of South Korea?. Kidney Blood Press. Res..

[11] Matsumoto A., Nagasawa Y., Yamamoto R., Shinzawa M., Hasuike Y., Kuragano T., Isaka Y., Nakanishi T., Iseki K., Yamagata K. (2017). The association of alcohol and smoking with CKD in a Japanese nationwide cross-sectional survey. Hypertens. Res..

[12] White S.L., Polkinghorne K.R., Cass A., Shaw J.E., Atkins R.C., Chadban S.J. (2009). Alcohol consumption and 5-year onset of chronic kidney disease: The AusDiab study. Nephrol. Dial. Transplant..

[13] Menon V., Katz R., Mukamal K., Kestenbaum B., de Boer I.H., Siscovick D.S., Sarnak M.J., Shlipak M.G. (2010). Alcohol consumption and kidney function decline in the elderly: Alcohol and kidney disease. Nephrol. Dial. Transplant..

[14] Koning S.H., Gansevoort R.T., Mukamal K.J., Rimm E.B., Bakker S.J., Joosten M.M. (2015). Alcohol consumption is inversely associated with the risk of developing chronic kidney disease. Kidney Int..

[15] Lin H.H., Chang H.Y., Chiang Y.T., Wu M.S., Lin J.T., Liao W.C. (2014). Smoking, drinking, and pancreatitis: A population-based cohort study in Taiwan. Pancreas.

[16] Lai Y.J., Hu H.Y., Lee Y.L., Ko M.C., Ku P.W., Yen Y.F., Chu D. (2018). Frequency of alcohol consumption and risk of type 2 diabetes mellitus: A nationwide cohort study. Clin. Nutr. (Edinb. Scotl.).

[17] Lai Y.J., Hu H.Y., Lin C.H., Lee S.T., Kuo S.C., Chou P. (2015). Incidence and risk factors of lower extremity amputations in people with type 2 diabetes in Taiwan, 2001–2010. J. Diabetes.

[18] Pan W.H., Lee M.S., Chuang S.Y., Lin Y.C., Fu M.L. (2008). Obesity pandemic, correlated factors and guidelines to define, screen and manage obesity in Taiwan. Obes. Rev..

[19] Ainsworth B.E., Haskell W.L., Whitt M.C., Irwin M.L., Swartz A.M., Strath S.J., O’Brien W.L., Bassett D.R., Schmitz K.H., Emplaincourt P.O. (2000). Compendium of physical activities: An update of activity codes and MET intensities. Med. Sci. Sports Exerc..

[20] Chiva-Blanch G., Urpi-Sarda M., Llorach R., Rotches-Ribalta M., Guillen M., Casas R., Arranz S., Valderas-Martinez P., Portoles O., Corella D. (2012). Differential effects of polyphenols and alcohol of red wine on the expression of adhesion molecules and inflammatory cytokines related to atherosclerosis: A randomized clinical trial. Am. J. Clin. Nutr..

[21] Wakabayashi I., Daimon T. (2015). Alcohol-independent beneficial cardiometabolic profile of individuals with hyper-HDL cholesterolemia in Japanese men and women. J. Clin. Lipidol..

[22] Huang S., Li J., Shearer G.C., Lichtenstein A.H., Zheng X., Wu Y., Jin C., Wu S., Gao X. (2017). Longitudinal study of alcohol consumption and HDL concentrations: A community-based study. Am. J. Clin. Nutr..

[23] Crandall J.P., Polsky S., Howard A.A., Perreault L., Bray G.A., Barrett-Connor E., Brown-Friday J., Whittington T., Foo S., Ma Y. (2009). Alcohol consumption and diabetes risk in the Diabetes Prevention Program. Am. J. Clin. Nutr..

[24] Kim S.H., Abbasi F., Lamendola C., Reaven G.M. (2009). Effect of moderate alcoholic beverage consumption on insulin sensitivity in insulin-resistant, nondiabetic individuals. Metab. Clin. Exp..

[25] Napoli R., Cozzolino D., Guardasole V., Angelini V., Zarra E., Matarazzo M., Cittadini A., Sacca L., Torella R. (2005). Red wine consumption improves insulin resistance but not endothelial function in type 2 diabetic patients. Metab. Clin. Exp..

[26] Baliunas D.O., Taylor B.J., Irving H., Roerecke M., Patra J., Mohapatra S., Rehm J. (2009). Alcohol as a risk factor for type 2 diabetes: A systematic review and meta-analysis. Diabetes Care.

[27] Tsubono Y., Yamada S., Nishino Y., Tsuji I., Hisamichi S. (2001). Choice of comparison group in assessing the health effects of moderate alcohol consumption. JAMA.

[28] Conigrave K.M., Hu B.F., Camargo C.A., Stampfer M.J., Willett W.C., Rimm E.B. (2001). A prospective study of drinking patterns in relation to risk of type 2 diabetes among men. Diabetes.

